# Influence of a Multiple Epoxy Chain Extender on the Rheological Behavior, Crystallization, and Mechanical Properties of Polyglycolic Acid

**DOI:** 10.3390/polym15132764

**Published:** 2023-06-21

**Authors:** Jianfeng Gao, Kai Wang, Nai Xu, Luyao Li, Zhao Ma, Yipeng Zhang, Kun Xiang, Sujuan Pang, Lisha Pan, Tan Li

**Affiliations:** 1 School of Materials Science and Engineering, Hainan University, Haikou 570228, China; 17889987289@163.com (J.G.); wang_kai126@163.com (K.W.); luyaoli1120@yeah.net (L.L.); taohua20042@163.com (Z.M.); zhangyipeng2013@163.com (Y.Z.); gzsfxyxk@163.com (K.X.); 2 School of Science, Hainan University, Haikou 570228, China; psjuan@hainanu.edu.cn; 3 School of Chemical Engineering and Technology, Hainan University, Haikou 570228, China; happylisap@hainanu.edu.cn; 4 Shiner National and Local Joint Engineering and Research Center, Shiner Industrial Co., Ltd., Haikou 570228, China; 13807679873@163.com

**Keywords:** polyglycolic acid, chain extender, rheology, non-isothermal crystallization, mechanical properties

## Abstract

This study investigated the impact of a multiple epoxy chain extender (ADR) on the rheological behavior, crystallization, and mechanical properties of polyglycolic acid (PGA). Tests of the torque and melt mass flow rate and dynamic rheological analysis were conducted to study the rheological behavior of PGA modified with ADR. The rheological results of the modified PGA showed a significantly increased viscosity and storage modulus with an increase in the ADR amount, which could be attributed to the chain extension/branching reactions between PGA and ADR. It was proved that ADR could be used as an efficient chain extender for tailoring the rheological performance of PGA. The Han plot of the modified PGA showed a transition of viscous behavior to elastic behavior, while the ADR content was increased from 0 to 0.9 phr. The formation of long-chain branches (LCBs) was confirmed via the Cole–Cole plot and weighted relaxation spectrum, wherein the LCBs substantially changed the rheological behavior of the modified PGA. The vGP plots predicted a star-type topological structure for the LCBs. The results of non-isothermal crystallization kinetics suggested that the crystallization of the modified PGA was predominantly homogeneous nucleation and three-dimensional growth. The crystallinity decreased slightly with the increase in the ADR amount. Compared to neat PGA, the modified PGA samples exhibited better tensile and flexural performances.

## 1. Introduction

People have turned their attention to biodegradable plastics due to the problem of environmental pollution caused by traditional plastic waste. This, in turn, has led to the rapid development of biodegradable plastics. The common biodegradable plastics currently available include poly(lactide) (PLA), polyhydroxyalkanoate (PHA), ply(butylene succinate) (PBS), poly(butylene-adipate-*co*-terephthalate) (PBAT), poly(*ε*-caprolactone) (PCL), polyglycolic acid (PGA), etc. [1,2,3,4,5,6,7]. In the future, biodegradable plastics are expected to replace traditional plastics for many applications, from food packaging, pharmaceuticals, and agriculture to textiles.

As one of the most famous biodegradable plastics, polyglycolic acid (PGA) can be made from either fossil-based natural gas or biomass (wood distillation, biomass gasification, and fermentation). PGA has gained increasing attention because of its excellent mechanical strength and stiffness, high heat resistance, and eco-friendly nature [7]. Meanwhile, its degradation in the natural environment is also faster than that of the other common biodegradable polyesters, mainly due to the high hydrophilicity of PGA and its hydrolysis in the absence of enzymes [7]. However, some shortcomings of PGA, such as its poor stability during thermal processing, narrow processing temperature window, and low melt strength, not only greatly limit its molding and processing but also reduce the service performance of PGA products.

It has been reported that PGA undergoes thermal degradation predominantly due to random scissions of the main chain [8]. This includes hydrolysis, ester interchange, the decarboxylation of terminal carboxyl groups, and thermo-oxidative degradation [9,10,11,12]. The thermogravimetric curve of PGA showed that thermogravimetric loss began to occur above 200 °C, and the temperature of the maximum weight loss rate was between 342 and 362 °C [8,9,13]. Depending on the temperature, one of the above-mentioned reactions is dominant, which is undesirable. For example, below 277 °C, the breakdown of PGA is primarily due to the ester interchange processes. Meanwhile, ester interchange at low temperatures is also accompanied by the decarboxylation of the terminal carboxyl groups of the polymer. Above 277 °C, the decomposition of PGA is mainly caused by random scissions of the main chain [9]. According to the literature, moisture can significantly enhance the thermal degradation of PGA [9,11]. In addition, the thermal degradation kinetics of PGA, synthesized through different synthetic routes (the solution-polymerized method and ring-opening polymerization method), were studied based on thermogravimetry, and the effects of end groups and molecular weight on the degradation were evaluated [14]. The results showed that the degradation of PGA involves random chain cession, regardless of the end groups and synthesis method.

The most common method of tackling the poor stability and low melt strength resulting from the thermal degradation of polyester materials, such as poly(lactide) (PLA), poly(butanediol terephthalate) (PBT), and poly(butylene-adipate-*co*-terephthalate) (PBAT) during processing and molding is to modify these polyesters with chain extenders (CEs). CEs, such as polycarbodiimide (PCD) [15,16], multi-functional epoxides [17,18,19], diisocyanates [20,21], dianhydrides [22,23], and tris(nonyl-phenyl) phosphate (TNPP) [24,25], increase the molecular weight and melt strength of polyesters. They also serve as reactive compatibilizers in the blend through the introduction of chain extension/branching reactions to reconnect the cracked chains. Among these, the multiple epoxy chain extender has the additional advantage of a wide processing window [26]. According to the literature, the multi-epoxide groups of CEs can theoretically react with both the terminal carboxyl groups and terminal hydroxyl groups of polyesters to undergo glycidyl esterification reaction and etherification reaction, respectively. This inevitably leads to the in situ generation of some chain-extended/-branched polyester macromolecules during melt processing [27,28]. Notably, in the case of polyesters, the reactivity of the carboxyl group with the epoxide group is higher than that of the hydroxyl group with the epoxide group [29,30,31,32]. This means that the reaction rate of the esterification of the terminal carboxyl group/epoxide group is faster than the reaction rate of the etherification of the terminal hydroxyl group/epoxide group, while the polyesters are modified with multiple epoxy CEs.

To date, there are only few literature reports on the effects of modification with CEs on the properties of PGA. Chen et al. [33] introduced two kinds of CEs into PGA, polystyrene-acrylonitrile styrene–acrylonitrile-glycidyl methacrylate terpolymer (poly(St-AN-GMA)) and 4, 4′-methylenebis(phenyl isocyanate) (MDI), which were effective in improving the strength and thermal stability of the PGA melt. Sun et al. [34] reported the modification of PGA through reactive extrusion using MDI as the CE and polycarbodiimide (PCDI) as an anti-hydrolysis agent to improve the thermal stability during processing and the rheological property of the PGA. It was found that upon the introduction of 3 wt% MDI and 1 wt% PCDI into the PGA matrix, the melt mass flow rate (MFR) drastically decreased from 37.8 g/10 min to 8.1 g/10 min. In addition, the accelerated hydrolysis aging test indicated that the hydrolysis rate constant of unmodified but one-pass-processed PGA at 30 °C was 0.06183 day^−1^, and that of the modified PGA was 0.01332 day^−1^, which exhibited a 78% reduction. Correspondingly, the service life of the modified PGA was significantly prolonged.

Although the above literature reported the influences of some kinds of chain extenders on the thermal stability and rheological behaviors of PGA, there are still some important and interesting scientific questions about the modification of PGA with multiple epoxy chain extenders that need to be studied in detail, such as (1) the formation and topological structure of long-chain-branched PGA, (2) the rheological responses of PGA upon the incorporation of the long-chain branches, and (3) the influences of multiple epoxy chain extenders on the other properties of PGA.

In this paper, polyglycolic acid (PGA) was modified with a multiple epoxy chain extender (BASF Joncryl ADR-4370s, abbreviated as ADR in this paper). The influence of ADR on the rheological behavior of PGA was studied by measuring the torque and melt mass flow rate and dynamic rheological analysis. The long-chain-branched structure of the modified PGA and its influence on the rheological response were characterized and analyzed using dynamic rheological analysis. In addition, differential scanning calorimetry and wide-angle X-ray diffractometry were used to study the impacts of the ADR content on non-isothermal crystallization kinetics and crystal structures of PGA. Additionally, the effects of the ADR content on the mechanical performance of the PGA were studied in tensile and flexural tests.

## 2. Materials and Methods

### 2.1. Materials

**Figure 1:**
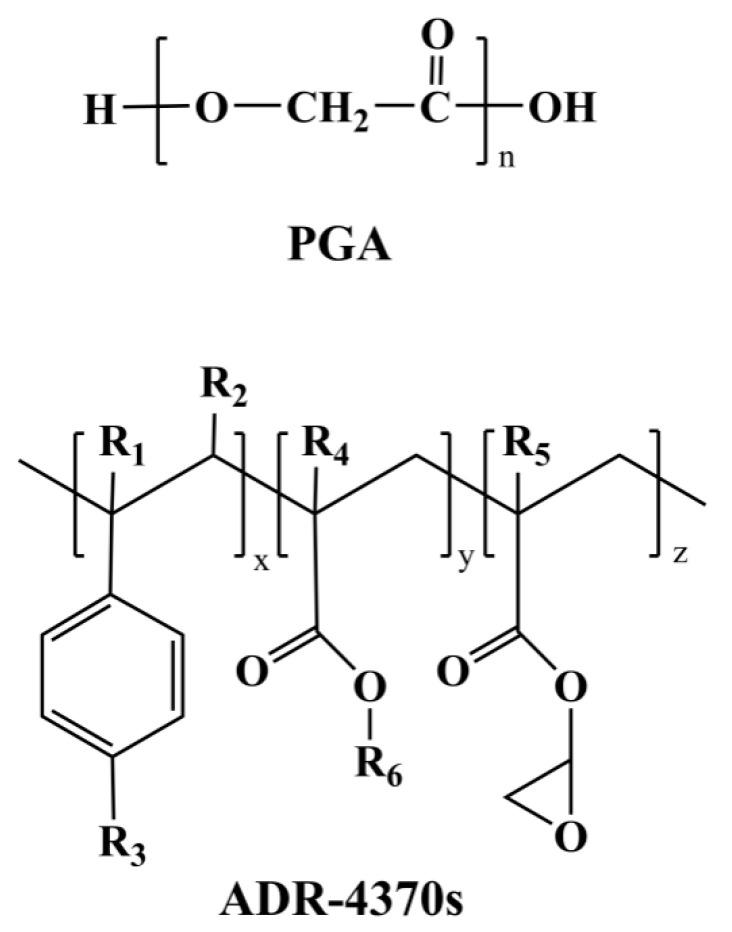
Structures of PGA and Joncryl ADR-4370s, where R1–R5 are H, CH_3_, a higher alkyl group, or combinations of them; R6 is an alkyl group, and x, y, and z are each between 1 and 20.

PGA, with a density of 1.53 g/cm^3^ and MFR of 14.7 g/10 min (235 °C, 2.16 kg), was obtained from Inner Mongolia Pujing Polymer Materials Technology Co. Ltd. (Bao Tou, China). The multiple epoxy chain extender (Joncryl ADR-4370s, abbreviated as ADR in this paper) was purchased from BASF SE (Ludwigshafen, Germany). Its molecular weight and epoxy equivalent weight were 6800 and 285 g/mol, respectively. The structures of the PGA and ADR are shown in Figure 1.

### 2.2. Sample Preparation

Prior to melt blending, the PGA was dried in a vacuum oven at 60 °C for 8 h. The dried PGA was compounded with ADR using an internal mixer (XS-60, Kechuang Rubber and Plastic Machinery Equipment Co. Ltd., Shanghai, China). The melt blending was conducted at 235 °C at a rotor speed of 50 rpm for 10 min. The PGA samples modified with different amounts of ADR were referred to as PGA_*x* (*x* = 0.3, 0.6, 0.9), where *x* denotes ADR parts per hundred weight of PGA resin. In addition, neat PGA was also processed under the same processing conditions for comparison.

Then, the neat PGA and modified PGA samples were injection-molded into specimens for tensile and flexural property testing using an injection-molding machine (WZS10D, Xinshuo Precision Machinery Co. Ltd., Shanghai, China). The temperatures of the upper cavity plate, the lower cavity plate, and the barrel of the injection-molding machine were 245, 245, and 230 °C, respectively. The screw speed was 75 rpm and the injection pressure was 0.4 MPa. Prior to testing, the specimens were stored at room temperature for 7 days.

### 2.3. Torque

The curves for the torque vs. mixing time of the neat PGA and modified PGA were plotted from the torque rheometry results (XSS-300, Kechuang Rubber & Plastic Equipment Co. Ltd., Shanghai, China). A steady temperature of 235.0 ± 1.0 °C was maintained, and the rotation speed was set as 50 rpm. The total mixing time was set as 30 min.

### 2.4. Melt Mass Flow Rate (MFR)

The MFRs of the samples were determined using an MFR apparatus (XNR-400B, Desheng Testing Equipment Co. Ltd., Chengde, China) at 235 °C using a load of 5.0 kg.

### 2.5. Dynamic Rheological Analysis

Dynamic rheological analysis was carried out with a rotary rheometer (Mars 60, Thermo Fisher Scientific, Karlsruhe, Germany) equipped with a parallel plate geometry and using 20 mm diameter plates. The samples with a thickness of 1.0 mm were melted at 235 °C in the fixture and then subjected to dynamic frequency sweep. The frequency sweep range was 0.1–628 rad/s, with a fixed strain of 1%.

### 2.6. Differential Scanning Calorimetry (DSC)

DSC thermograms were acquired using a DSC instrument (Q100, TA Instruments, New Castle, DE, USA). In the non-isothermal crystallization runs, the sample (5–8 mg) was quickly heated to 240 °C at 50 °C/min. The samples were held at 240 °C for 3 min in order to completely erase the thermal history. This was followed by cooling down to 20 °C at different cooling rates (5/10/15/20 °C/min). Additionally, the samples were held at 20 °C for 3 min. Finally, the samples were re-heated from 20 to 240 °C at a heating rate of 10 °C/min.

In addition to the testing of non-isothermal crystallization behavior, the crystallinities ($X_{c}$) of the injection-molded samples were also determined. Each sample, weighing 5–8 mg, was heated from 20 to 240 °C at a heating rate of 10 °C/min. All runs were carried out in a nitrogen (N_2_) atmosphere. The relevant parameters were determined from the DSC thermograms, and $X_{c}$ of the PGA component was calculated according to Equation (1):

(1) $$X_{c}=\frac{\left|\Delta H_{m}\right|}{\omega \times \left|\Delta H_{m}^{0}\right|}\times 100\%$$

where $\Delta H_{m}$ is the melting enthalpy of the PGA component, ω is the mass fraction of the PGA component, and $\Delta H_{m}^{0}$ is the ideal melting enthalpy (−198.15 J/g [35]) of 100% crystalline PGA.

### 2.7. Wide-Angle X-ray Diffraction (WAXD) Analysis

The crystalline structures of the samples were determined using WAXD (Smart Lab, Rigaku Co., Akishima, Japan). A Cu Kα radiation source (λ = 1.542 Å) was used at a 40 kV voltage and 40 mA current. The WAXD data were recorded in the 2θ range of 5–35° at a scanning speed of 2°/min.

### 2.8. Tensile and Flexural Testing

An electronic universal testing machine (WSW-2KN, Changchun Intelligent Instrument Equipment Co., Ltd., Changchun, China) was used to evaluate the tensile and flexural performances of the samples.

The tensile yield strength and elongation at break were determined at a crosshead velocity of 5 mm/min, whereas the tensile modulus was determined at a crosshead velocity of 1 mm/min, equipped with a small-gauge-length extensometer. The flexural strength and flexural modulus were measured at a crosshead velocity of 1 mm/min, and the deflection was 6 mm. The average value was calculated from at least five valid values for each data set, along with the standard deviation (SD).

## 3. Results and Discussion

### 3.1. Rheological Investigation

**Figure 2:**
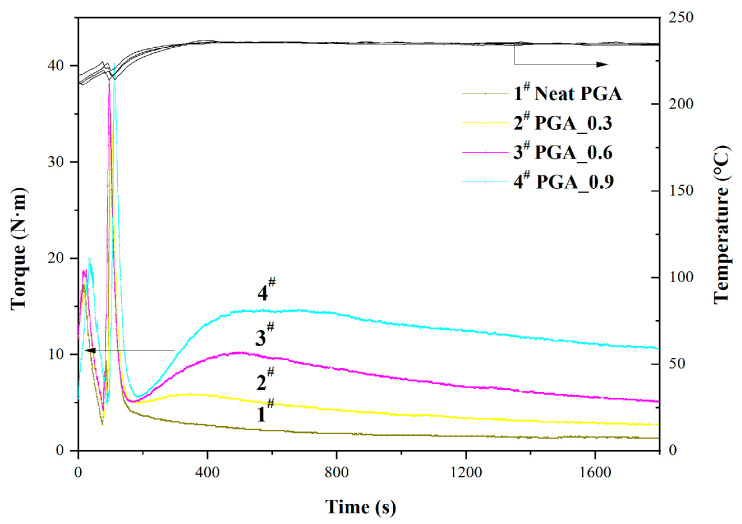
Curves for torque values of the neat PGA and modified PGA samples at 235 °C.

The torque values, as a function of time, were measured for the neat PGA and modified PGA, as shown in Figure 2. In addition, the plots of the melt temperature vs. blending time are also shown in Figure 2. The desired experimental temperature of the melts in the blending system was kept at 235.0 ± 1.0 °C.

**Scheme 1:**
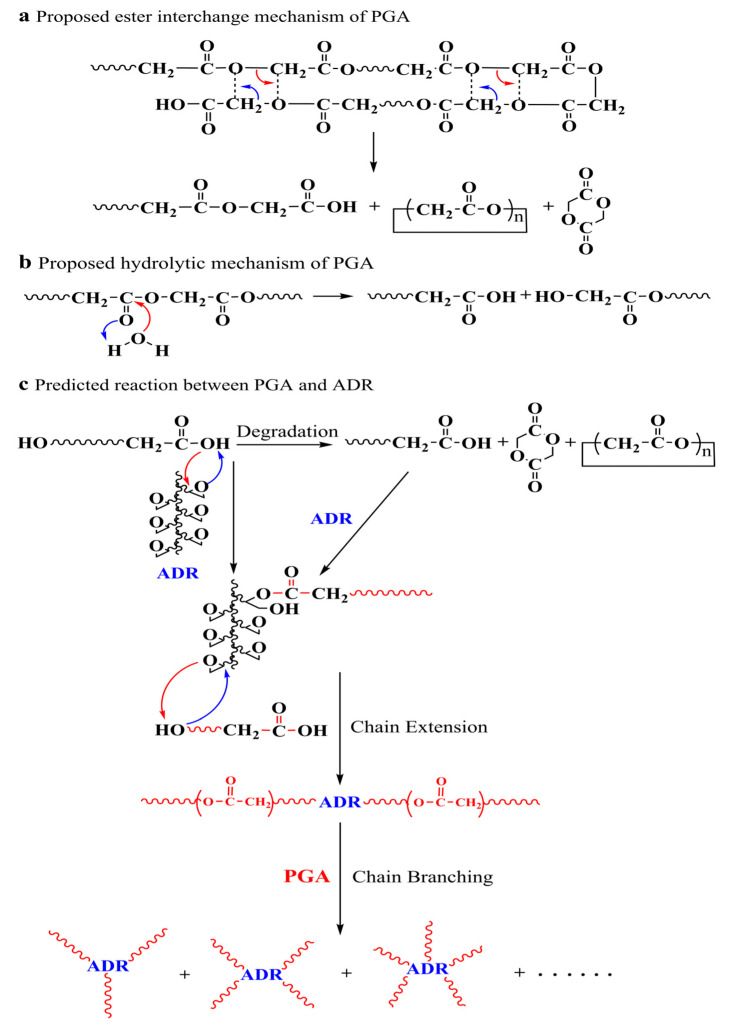
Proposed mechanisms of PGA degradation and predicted chain extension/branching reactions between PGA and ADR.

As seen in Figure 2, the torque plots of the neat PGA and modified PGA showed strong torque peaks from 0 s to approximately 180 s, which corresponded to the feeding and melting processes of PGA granules. While the mixing time reached approximately 180 s, the PGA granules completely melted. With the further prolonging of the mixing time from 180 s to 1800 s, the neat PGA showed a decline in its torque value, therefore highlighting the occurrence of thermal degradation during the mixing process. McNeill studied the isothermal degradation of PGA at different temperatures between 230 °C and 440 °C via thermal volatilization analysis (TVA) [9]. Below 277 °C, ester interchange was the main mechanism of thermal degradation of the PGA. Ester interchange at the terminal or internal positions of PGA macromolecular chains produces glycolides and larger cyclic oligomers. In particular, the hydrolysis of polyester cannot be ignored. The plausible mechanisms of ester interchange and hydrolysis for PGA are proposed in Scheme 1a,b. As a comparison, the plot for the torque values of the modified PGA showed a significant ascent with the increase in the ADR content. This indicated that the ADR could effectively improve the melt viscosity of PGA through chain extension. Theoretically, the epoxide groups of the ADR can react with both the hydroxyl and carboxyl end groups of the polyesters to form high-molecular-weight linear PGA or even long-chain-branched (LCB) PGA. Notably, studies have shown that in the reaction between polyester and epoxide, the formation of glycidyl esters of carboxyl acid end groups was more prominent than the etherification of hydroxyl end groups [29]. Based on these studies, a plausible mechanism for the chain extension/branching reactions between PGA and ADR in our study was proposed, as shown in Scheme 1c. It demonstrated that the chain extension/branching reactions not only hampered the thermal degradation of PGA but also drastically increased the melt viscosity of PGA due to the formation of extended PGA and long-chain-branched PGA.

The flow properties of the neat PGA and modified PGA were evaluated by testing their melt mass flow rates (MFR), as shown in Table 1.

As seen in Table 1, the MFR of the processed neat PGA was as high as 54.76 g/10 min. However, with increase in the ADR content, the MFR of the modified PGA samples showed a pronounced decrease. For example, when the ADR content was increased to 0.9 phr, the MFR of PGA_0.9 decreased drastically to 0.51 g/10 min. This indicated that the ADR content had a very obvious effect on the PGA’s melt viscosity, which was also consistent with the torque results.

**Figure 3:**
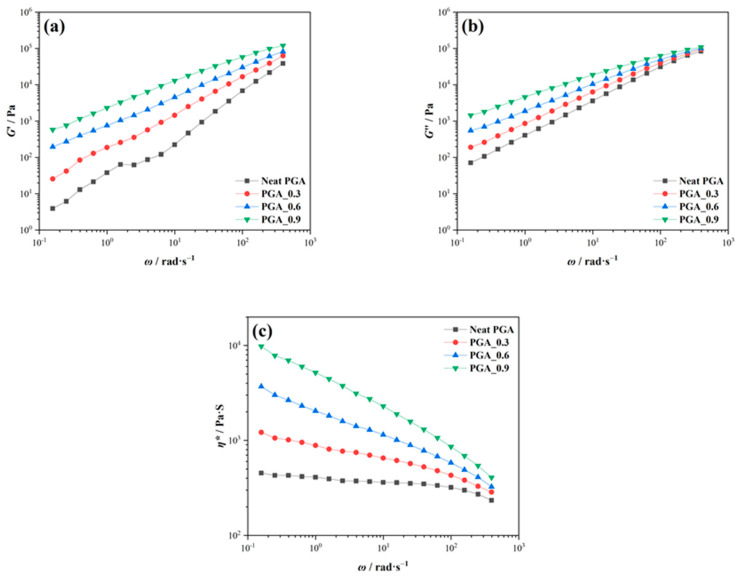
Rheological behaviors of neat PGA and modified PGA: (**a**) storage modulus; (**b**) loss modulus; (**c**) complex viscosity.

The dynamic rheological behaviors of the neat PGA and modified PGA were further characterized and analyzed using a rotary rheometer. Figure 3a–c shows the storage modulus ($G^{\prime }$), loss modulus ($G^{\prime \prime }$), and complex viscosity (η*) curves of the neat PGA and modified PGA, respectively.

It is evident from Figure 3a,b that with addition of ADR, the storage modulus $G^{\prime }$ and loss modulus $G^{\prime \prime }$ of the modified PGA increased significantly. This could be attributed to the chain extension/branching reactions between the PGA and ADR. The presence of a large number of branched structures, in addition to linear extended ones, increased the entanglement between the PGA chains, resulting in a pronounced increase in the strength and elasticity of the polymer melt [30]. In Figure 3c, the neat PGA shows a stable Newtonian plateau in the middle- and low-frequency regions and only obvious shear thinning in the high-frequency region. It is noteworthy that the addition of ADR effectively improved the complex viscosity of the PGA. With increase in the ADR content, the complex viscosity of the modified PGA increased gradually at a low frequency. Furthermore, shear thinning became obvious at a lower frequency. The complex viscosity of the modified PGA became more sensitive to the changes in frequency with the further increase in the ADR content. According to the literature, the Newtonian plateau shifts to a lower frequency or even disappears with the increase in molecular weight or with long-branched chain structures [36]. It was obvious that the degree of chain extension/branching drastically increased as the ADR content was increased. The use of a chain extender ADR increased the viscosity, melt strength, and moduli of the modified PGA. It facilitated further processing, since a high melt viscosity and elasticity are required in processes such as extrusion, blown film processing, vacuum forming, and foaming.

**Figure 4:**
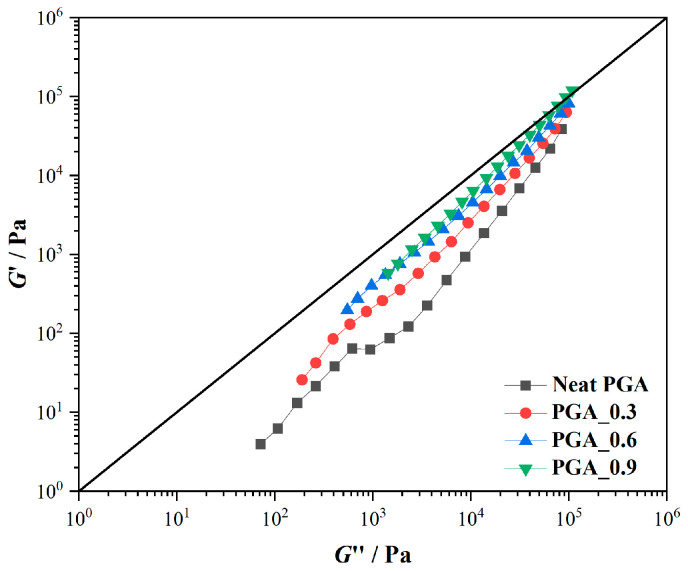
Han plots of neat PGA and modified PGA.

The viscoelastic transition of a polymer melt can be represented by the line of $G^{\prime }=G^{\prime \prime }$ in Han plots, where $G^{\prime }$ and $G^{\prime \prime }$ represent the typical elastic and viscous properties, respectively. The Han plots of the modified PGA are shown in Figure 4.

In Figure 4, the curves of the modified PGA gradually approach the line of $G^{\prime }=G^{\prime \prime }$ with the addition of ADR in the low-frequency region. In other words, the storage modulus increased under the same loss modulus. This indicated that the modified PGA rapidly changed from viscous behavior to elastic behavior. This could be attributed to the great formation of chain-extended and -branched PGA chains.

**Figure 5:**
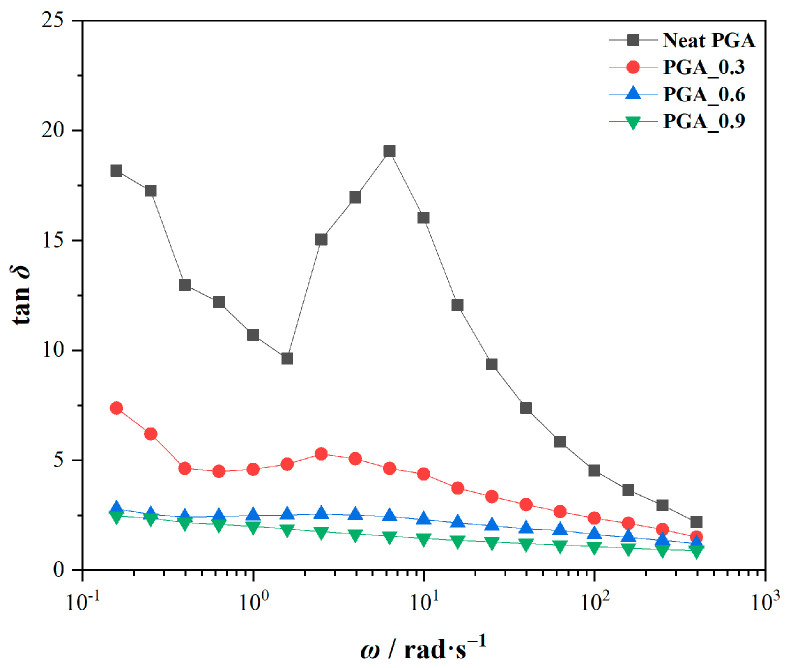
Loss tangent of neat PGA and modified PGA versus angular frequency.

The loss angle (δ) of a viscoelastic fluid is also known as the phase angle, which is the phase difference between the strain and stress of the viscoelastic fluid under external force due to the joint actions of viscosity and elasticity, $\tan\;\delta =G^{\prime }/G^{\prime \prime }$. The tan δ−ω curves of the neat PGA and modified PGA are shown in Figure 5.

As seen from Figure 5, the modified PGA, compared to the neat PGA, showed a decrease in tan δ at low frequencies, which corresponded to an increase in the terminal relaxation time. For polymer melts, the relaxation time is a function of the relative molecular weight of the polymer and the branching of the long chain [37,38,39]. In particular, when the ADR content increased to 0.9 phr, the tan δ plot gradually turned into a straight line in the scanning range. Some studies have shown that polymers containing long-chain branches (LCB) showed gel-like rheological behaviors, that is, the *δ* or tan *δ* was independent of the frequency in a limited range [40,41].

**Figure 6:**
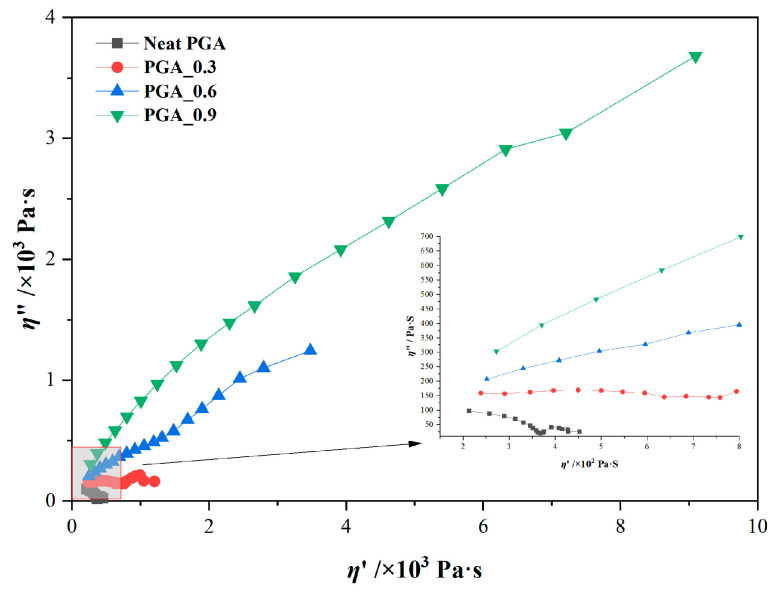
Cole–Cole plots of neat PGA and modified PGA.

The formation of branched structures and the degree of branching can be demonstrated using a Cole–Cole plot [42,43,44]. The Cole–Cole plots of the neat PGA and modified PGA are shown in Figure 6, where $\eta^{\prime }\;(\eta^{\prime }=G^{\prime \prime }/\omega )$ and $\eta^{\prime \prime }\;(\eta^{\prime \prime }=G^{\prime }/\omega )$ are the real and imaginary parts of η*, respectively.

The resemblance of the Cole–Cole plot, for a linear polymer, is closer to a semicircle, and the higher the molecular weight is, the greater its radius is [44]. However, for neat PGA, the Cole–Cole plot showed an irregular semicircle, which could be the reason for its low viscosity [42]. With the increase in the ADR content, the radius of the Cole–Cole plot for the modified PGA greatly increased and showed a more obvious upturn at a high viscosity, which corresponded to a lower frequency. This indicated that there were many branched structures in addition to the linear ones. Further, the higher the degree of branching was, the greater the upturn was [44].

**Figure 7:**
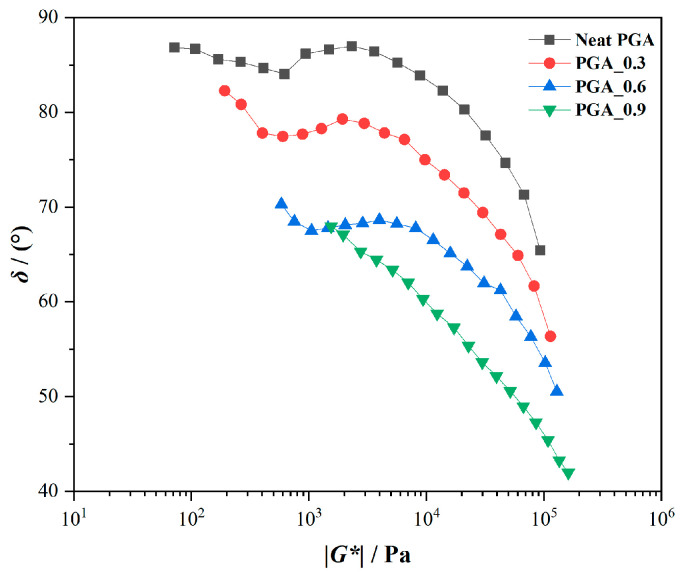
vGP plots of neat PGA and modified PGA.

In order to better understand the topological structures of the neat PGA and modified PGA, the van Gurp–Palmen (vGP) plots of these samples are shown in Figure 7.

It can be seen from Figure 7 that the loss angle, δ, gradually decreased as the absolute value of the complex modulus $│G*│\;(│G*│{=(G^{\prime }}^{2}+{G^{\prime \prime }}^{2}{)}^{1/2})$ increased. In particular, for the neat PGA, δ was nearly 90°, which implied that the samples were almost completely viscous at low values of │G*│. However, as the ADR content increased, the vGP plot of the modified PGA gradually showed deviation from that of the neat PGA. Lohse [45] found that the star-shaped and comb-shaped branched structures could be distinguished from the vGP plots of the branched polymer. For the star-shaped topological structure, the difference in the area under the curves can be related to the degree of branching. The rheological characteristics appearing in the vGP plots for the modified PGA are very similar to those for the star-shaped polymer reported in the literature [45]. Hence, based on the characteristics of chain extension/branching reactions and the vGP plot, it could be speculated that the branched structures of modified PGA should be closer to a star-shaped topological structure.

**Figure 8:**
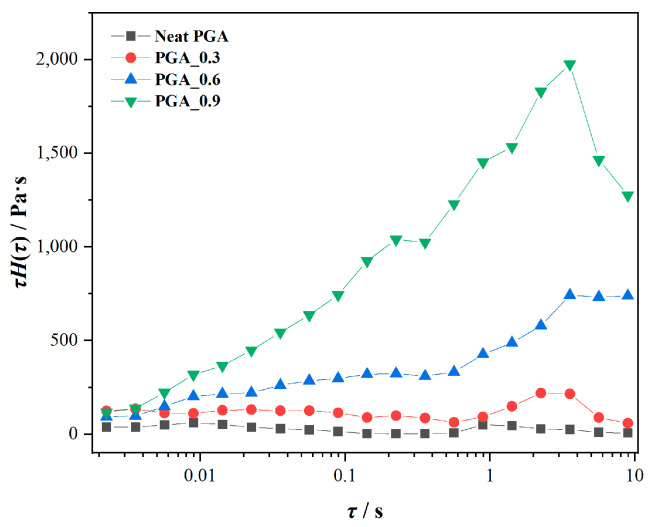
Weighted relaxation spectra of neat PGA and modified PGA.

In addition, the weighted relaxation spectra τH(τ) for the neat PGA and modified PGA samples are presented in Figure 8.

The neat PGA showed only one relaxation peak, which corresponded to this homogeneous component. However, in the case of the modified PGA samples, two main relaxation times were clearly observed, which could be interpreted as the simultaneous occurrence of two relaxation processes. The higher value of the relaxation time corresponded to the relaxation of the long-chain branch (LCB) structures, wherein these LCB structures would pronouncedly restrict the chain mobility and retard the overall relaxation of the modified PGA. Furthermore, the lower relaxation time was consistent with the relaxation of the short chains of PGA. The same double time relaxation distribution was also observed by Wood-Adams and Costeux for long-chain-branched PE [46].

### 3.2. DSC and WAXD Analyses

The non-isothermal crystallization behavior and non-crystallization kinetics of the neat PGA and modified PGA at different cooling rates (Φ) were studied via DSC. The DSC thermograms showing the cooling and subsequent heating of the samples are presented in Figures S1 and S2, respectively. The onset temperature of crystallization ($T_{o}$), peak temperature of crystallization ($T_{p}$), end temperature of crystallization ($T_{e}$), temperature range for complete crystallization ($\Delta T=T_{o}-T_{e}$), and the time required for complete crystallization ($t_{c}=\Delta T$/Φ) at different cooling rates (Φ) are listed in Table 2. Furthermore, the melting temperature ($T_{m}$) and melting enthalpy ($\Delta H_{m}$) in the second heating run of the DSC analysis, along with the corresponding degrees of crystallinity ($X_{c}$) of the samples, are also presented in Table 2.

For the neat PGA and modified PGA, $T_{o}$, $T_{p}$, and $X_{c}$ all decreased with the increase in the cooling rate. This was expected, since the mobility of the macromolecular chains and segments was strongly dependent on the temperature and cooling rate of the crystallization process. When the cooling rate was too fast, the chain folding of the PGA macromolecules was severely restricted, resulting in a delayed crystallization process and reduced crystallinity. Moreover, at the same cooling rate, $T_{o}$, $T_{p}$, and $X_{c}$ also decreased as the ADR content increased from 0 to 0.9 phr. This was due to the fact that the regularity of the PGA chains and segments were disturbed by the chain extension and branched structures. Interestingly, with the increase in the ADR content, the time required for complete crystallization ($t_{c}$) showed an overall gradual decrease. This implied that the crystallization rate of the modified PGA increased upon the addition of ADR. The influence of the ADR content on the crystallization process is considered to be relatively more complex. On the one hand, the chain-extended and -branched structures decreased the regularity of the PGA chains and segments and thus led to a decrease in $T_{o}$ and $T_{p}$. On the other hand, however, an increased melt viscosity of the modified PGA was conductive to increasing the nucleation density [47,48,49]. Consequently, the higher nucleation density increased the crystal growth rate. As a result, the overall crystallization rate increased to some extent when ADR was added. Earlier studies also reported that the addition of a multiple epoxy chain extender can increase the melt crystallization rate of PLA [47,48,49].

The effect of the ADR content on the non-isothermal crystallization kinetics of PGA was studied further. In the study of the non-isothermal crystallization of neat PGA and modified PGA, the time of the beginning of PGA crystallization was assumed to be $t_{o}$, and the temperature at this time was $T_{o}$. Similarly, $t_{e}$ and $T_{e}$ represent the time and temperature for complete crystallization. The relation between the relative crystallinity X(T) and crystallization temperature T is shown in Equation (2):

(2) $$X(T)=\frac{{ʃ}_{T_{0}}^{T}{(dH}_{c}/dT)dT}{{ʃ}_{T_{0}}^{T_{e}}{(dH}_{c}/dT)dT}$$

**Figure 9:**
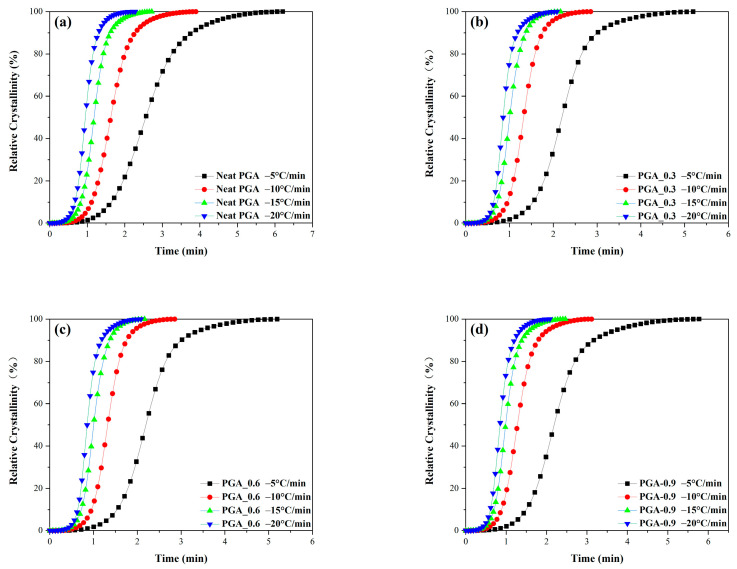
Relationship between relative crystallinity (X(t)) and crystallization time (t). (**a**) Neat PGA; (**b**) PGA_0.3; (**c**) PGA_0.6; and (**d**) PGA_0.9.

The relative crystallinity X(t), as a function of crystallization time t, can be obtained through the transformation of Equation (3):

(3) $$t=\frac{T_{0}\;-\;T}{\Phi }$$

where T is the crystallization temperature at time t, and Φ is the cooling rate. Figure 9 shows the relative crystallinity curves of the neat PGA and modified PGA as a function of time at different cooling rates.

All the curves showed similar sigmoid dependence with the crystallization time. In addition, the higher the cooling rate was during crystallization, the faster the crystallization rate of the neat PGA and modified PGA was.

**Figure 10:**
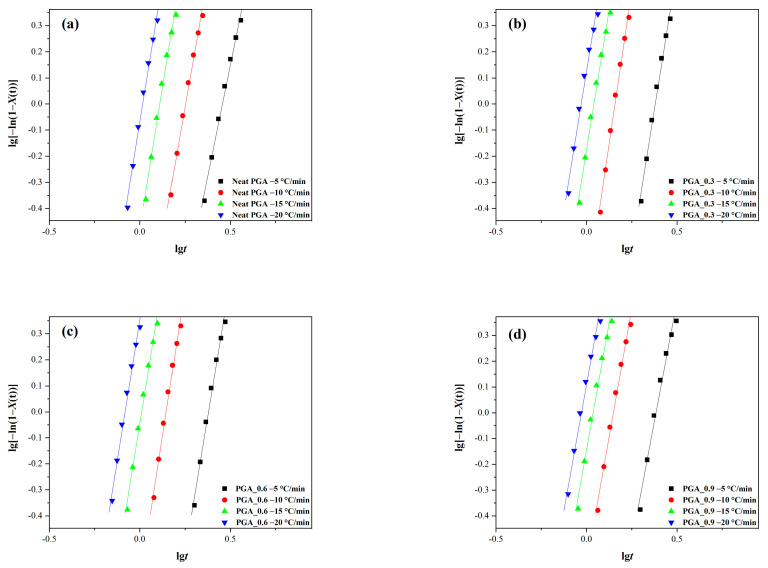
Linear fitting plots of lg[−ln(1−X(t))] and lgt. (**a**) Neat PGA; (**b**) PGA_0.3; (**c**) PGA_0.6; and (**d**) PGA_0.9.

The Avrami equation (Equation (4)) was originally a theory of crystallization kinetics in the process of metal crystallization and was gradually popularized for the isothermal crystallization kinetics of polymers:

(4) lg[−ln(1−X(t))]=nlgt+lgK

where n is the Avrami exponent that depends on the nature of nucleation and growth geometry of the crystals, and K is the crystallization rate constant, including both nucleation and growth rate parameters. Further research showed that the Avrami equation was only suitable for describing the isothermal crystallization behaviors of polymers in the initial stage. However, for the treatment of non-isothermal crystallization processes, the physical meaning of the kinetic parameters was not clear [50]. Jeziorny showed that the non-isothermal crystallization process of polymers could be studied using the Avrami equation after the crystallization rate constant (K) was modified with the non-isothermal crystallization kinetic constant ($K_{c}$) [51]. The relationship between $K_{c}$ and *K* is shown in Equation (5):

(5) $${\mathrm{lg}K}_{c}=\frac{\mathrm{lg}K}{\Phi }$$

where *Φ* stands for cooling rate. The linear fitting plots of lg[−ln(1−X(t))] and lgt for the neat PGA and modified PGA at different cooling rates are presented in Figure 10. It was evident that lg[−ln(1−X(t))] had a good linear correlation with lgt.

The corresponding linear fitting parameters are presented in Table 3. The exponent n is a constant, the value of which depends on the type of nucleation and growth process parameters. The value of n was approximately 4, which implied that the crystallization of PGA with and without ADR under non-isothermal conditions was a homogeneous nucleation and three-dimensional growth process. In addition, at the same cooling rate, $\mathrm{lg}K_{c}$ generally increased with the increase in the ADR content, which was consistent with the trend seen for the ADR content at time $t_{c}$ for the completion of crystallization (see Table 2).

**Figure 11:**
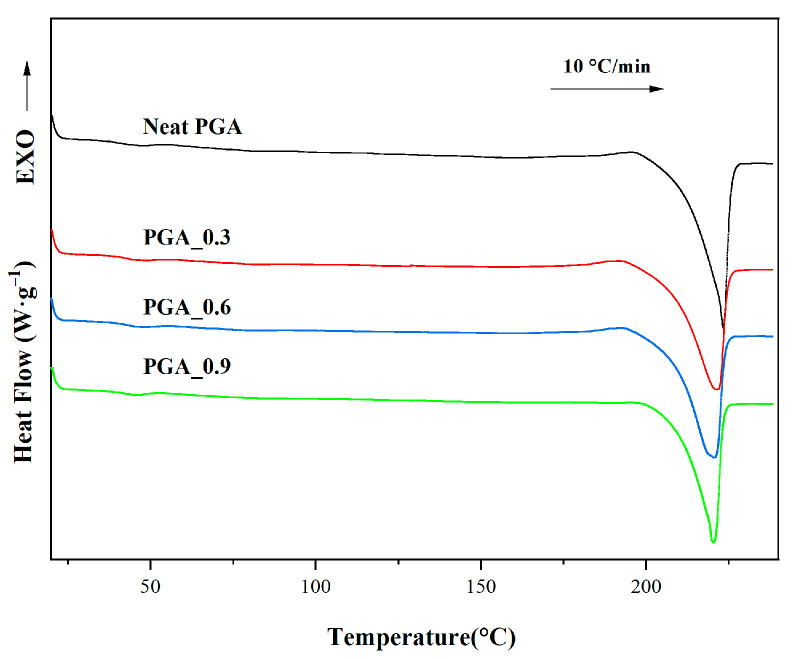
First heating DSC thermograms of the injection-molded samples of neat PGA and modified PGA.

Furthermore, the crystalline properties and structures of the injection-molded samples of neat PGA and modified PGA were characterized using DSC and WAXD measurements. The DSC thermograms showing the first heating of the injection-molded samples are shown in Figure 11, and the glass transition temperature ($T_{g}$), $T_{m}$, $\Delta H_{m}$, and $X_{c}$ are listed in Table 4.

As shown in Table 4, $T_{m}$ decreased as the ADR content increased. The dependence of lamellar crystal thickness $l_{c}$ and $T_{m}$ could be expressed using Equation (6) [52]:

(6) $$l_{c}=\frac{{2\;\sigma }_{e}}{\Delta H_{m}^{0}\;\rho \;(1-\frac{T_{m}}{T_{m}^{0}})}$$

where $\sigma_{e}$ is the free energy of the folded lamellar surface of PGA, ρ is the density of PGA, and $T_{m}^{0}$ is the equilibrium melting peak temperature of PGA. According to Equation (6), the lower the $T_{m}$ is, the smaller is the $l_{c}$ is. As can be seen in Table 4, the $T_{m}$ of the modified PGA decreased with the increase in the ADR content, indicating a continuous decrease in lamellar crystal thickness. At the same time, the crystallinity $X_{c}$ of the modified PGA also showed the same trend. This was mainly attributed to the disturbance of the regularity of the PGA chains and segments by the chain extension/branching structures, which resulted in incomplete crystal development.

**Figure 12:**
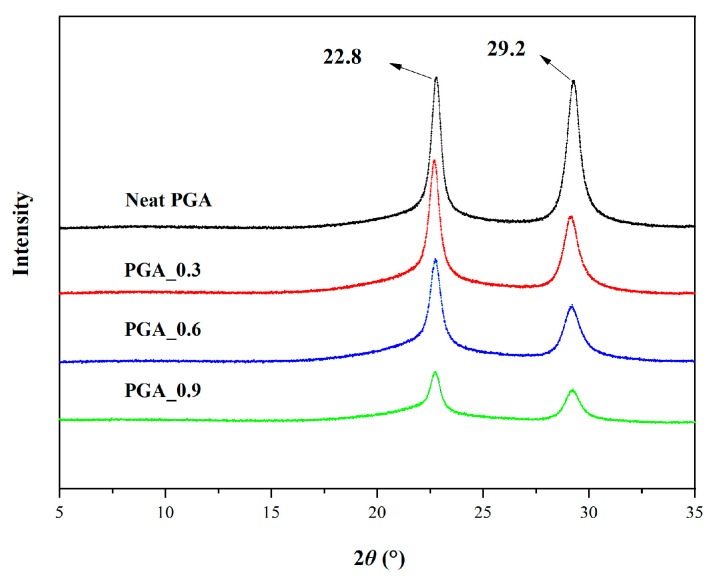
WAXD patterns of the injection-molded samples of neat PGA and modified PGA.

The WAXD analysis was conducted to understand the impact of the ADR content on the PGA crystal structure. Figure 12 presents the WAXD patterns of the neat PGA and modified PGA in the 2θ range of 5–35°.

In Figure 12, the strong diffraction peaks at 22.8° and 29.2° corresponded to the (110) and (020) crystal planes of the neat PGA, respectively [14,53,54]. With the increase in the ADR content, the WAXD profile showed no new diffraction peaks or obvious peak shifting. However, the intensities of the diffraction peaks showed gradual decrease, indicating that the addition of ADR decreased the crystallinity of the PGA. This was consistent with the DSC results of the injection-molded specimens.

### 3.3. Mechanical Properties

**Figure 13:**
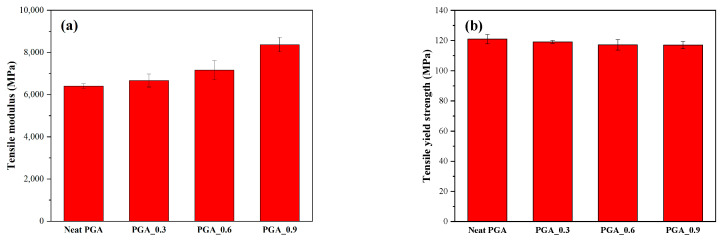

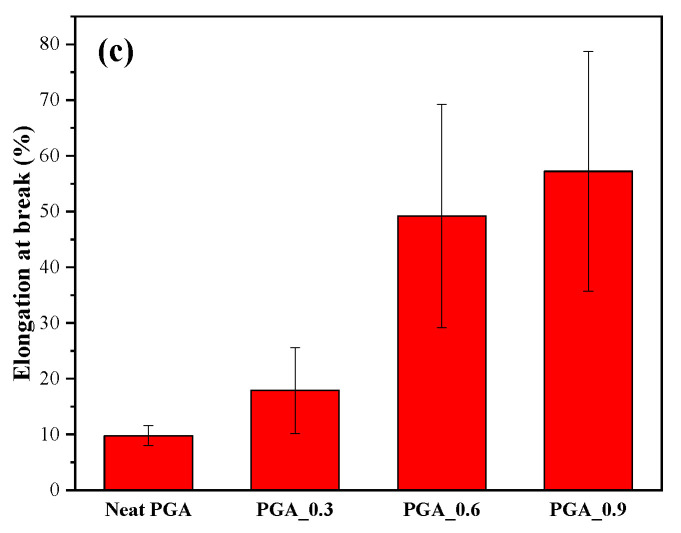
Tensile properties of the injection-molded samples of neat PGA and modified PGA. (**a**) Tensile modulus. (**b**) Tensile yield strength. (**c**) Elongation at break.

The tensile properties of the neat PGA and modified PGA are summarized in Figure 13, and their typical tensile stress–strain curves are shown in Figure S3.

With the increase in the ADR content, the tensile modulus increased from 6412.10 MPa for the neat PGA to 8372.40 MPa for PGA_0.9. The improvement in the deformation resistance of the PGA could be due to the fact that the extended/branched PGA macromolecules were more prone to entanglement, and thus the number of entanglement nodes in the PGA macromolecular chains and segments in the amorphous region was significantly increased. In fact, the increase in the number of entanglement nodes is also the main reason for the increased viscosity of the modified PGA melts. Notably, the PGA_0.6 and PGA_0.9 samples achieved 49.2% and 57.2% elongation at break, respectively, which were higher than the value for neat PGA (9.8%). The impact of the toughening mechanism of adding ADR on the tensile properties of PGA needs to be studied further.

**Figure 14:**
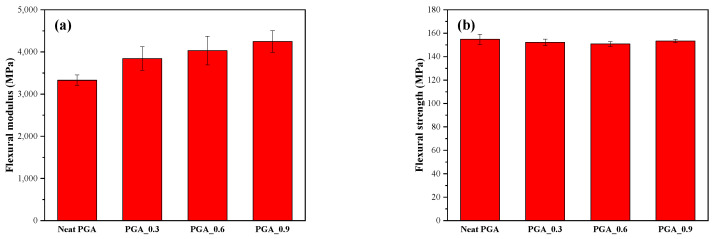
Flexural properties of the injection-molded samples of neat PGA and modified PGA. (**a**) Flexural modulus. (**b**) Flexural strength.

In addition, the flexural properties of the neat PGA and modified PGA are summarized in Figure 14, and their typical flexural load–deformation curves are shown in Figure S4.

When the ADR content increased to 0.9 phr, the flexural modulus of the modified PGA increased to 4246.22 MPa, which was 27.6% higher than that of the neat PGA (3329.04 MPa). This was consistent with the effect of adding ADR on the tensile modulus of modified PGA samples.

## 4. Conclusions

The effect of the ADR content on the rheological behavior of PGA was characterized using torque and MFR measurements and rotary rheometry. The plots of the torque testing showed that the torque of the modified PGA blending system increased with the increase in the ADR content. This could be attributed to the chain extension/branching reactions between the PGA and ADR. It showed that ADR could serve as an efficient chain extender to modify the rheological behavior of PGA. The results of the MFR and rotary rheometry measurements showed that the modified PGA exhibited a higher complex viscosity and higher storage modulus. This increase became more pronounced as the ADR content increased. Furthermore, the rheological investigations confirmed that the modified PGA showed rapid changes from viscous behavior to elastic behavior with the addition of ADR. This elastic rheological behavior of the modified PGA could mainly be attributed to the great formation of long-chain branches due to the in situ reaction between PGA and ADR. In contrast to the neat PGA, the modified PGA samples exhibited two relaxation processes in their weighted relaxation spectra. The higher value of the relaxation time corresponded to the relaxation of the long-chain branch (LCB) structures, wherein these LCB structures significantly restricted the chain mobility and retarded the overall relaxation of the modified PGA. In addition, the vGP plots showed the LCB structures to be closer to a star-type topological structure.

The non-isothermal crystallization kinetics and crystalline structure of the neat PGA and modified PGA samples were studied via DSC and WAXD. According to the Avrami–Jeziorny method, the values of exponent n for the neat PGA and modified PGA were around 4. This indicated that the PGA crystals were dominated by homogeneous nucleation and three-dimensional growth. Compared with the neat PGA, the modified PGA showed a slight decrease in the crystallization temperature and crystallinity with the increase in the ADR content. However, it is worth mentioning that the addition of ADR appeared to be useful in improving the crystallization rate of PGA to some extent.

The results of mechanical testing showed that the addition of ADR improved the stiffness and tensile ductility of PGA. When the ADR content was increased from 0 to 0.9 phr, the tensile modulus and flexural modulus increased from 6412.10 MPa and 3329.04 MPa for neat PGA to 8372.40 MPa and 4246.22 MPa for PGA_0.9, respectively. In addition, the elongation at break also increased from 9.8% to 57.2% as the ADR content increased from 0 to 0.9 phr.

## Figures and Tables

**Table 1:** MFRs of neat PGA and modified PGA.

Samples	MFR (g/10 min)
Neat PGA	54.76 ± 0.28
PGA_0.3	9.74 ± 0.77
PGA_0.6	0.87 ± 0.20
PGA_0.9	0.51 ± 0.07

**Table 2:** Thermal parameters of neat PGA and modified PGA.

Samples	*Φ* (°C/min)	*T*_o_ (°C)	*T*_p_ (°C)	*T*_e_ (°C)	Δ*T* (°C)	*T*_c_ (min)	*T*_m_ (°C)	Δ*H*_m_ (J/g)	*X*_c_ (%)
Neat PGA	−5	205.4	198.3	192.2	13.2	2.64	220.0	−91.49	46.2
	−10	202.0	194.4	187.9	14.1	1.41	220.6	−89.03	44.9
	−15	198.2	191.5	184.4	13.8	0.92	221.8	−84.33	42.6
	−20	197.0	190.0	182.3	14.7	0.74	222.0	−80.83	40.8
PGA_0.3	−5	200.5	196.2	190.0	10.5	2.10	218.8	−87.38	44.2
	−10	197.2	192.4	187.0	10.2	1.02	218.3	−84.07	42.6
	−15	195.5	190.2	184.0	11.5	0.77	219.9	−79.97	40.5
	−20	193.3	188.1	181.5	11.8	0.59	219.4	−75.86	38.4
PGA_0.6	−5	199.5	194.9	190.5	9.0	1.80	218.2	−84.18	42.7
	−10	196.2	191.5	186.5	9.7	0.97	216.9	−78.50	39.9
	−15	194.7	189.7	183.7	11.1	0.74	217.2	−76.21	38.7
	−20	193.0	188.1	181.8	11.2	0.56	219.0	−74.86	38.0
PGA_0.9	−5	199.5	194.8	190.4	9.1	1.82	218.0	−81.69	41.6
	−10	196.5	191.1	185.9	10.6	1.06	217.4	−76.17	38.8
	−15	193.6	188.2	181.9	11.7	0.78	217.5	−74.01	37.7
	−20	192.2	186.1	178.6	13.6	0.68	218.2	−71.88	36.6

**Table 3:** Non-isothermal crystallization parameters calculated using the Avrami–Jeziorny method.

*Φ* °C/min	Neat PGA			PGA_0.3			PGA_0.6			PGA_0.9		
	n	lgK	${\mathrm{lg}K}_{c}$	n	lgK	${\mathrm{lg}K}_{c}$	n	lgK	${\mathrm{lg}K}_{c}$	n	lgK	${\mathrm{lg}K}_{c}$
−5	3.4	−1.552	−0.310	4.3	−1.643	−0.329	4.2	−1.597	−0.319	3.7	−1.434	−0.287
−10	4.0	−1.005	−0.101	4.7	−0.739	−0.074	4.5	−0.662	−0.066	4.0	−0.591	−0.059
−15	4.3	−0.478	−0.032	4.3	−0.176	−0.012	4.3	−0.047	−0.003	4.0	−0.145	−0.010
−20	4.3	−0.070	−0.003	4.3	0.125	0.006	4.3	0.351	0.018	4.0	0.109	0.005

**Table 4:** Thermal parameters of the injection-molded samples of neat PGA and modified PGA.

Samples	*T*_g_ (°C)	*T*_m_ (°C)	Δ*H*_m_ (J/g)	*X*_c_ (%)
Neat PGA	41	223.3	−74.79	37.7
PGA_0.3	43	221.3	−71.00	35.9
PGA_0.6	43	220.6	−69.14	35.1
PGA_0.9	43	220.4	−60.19	30.6

## Data Availability

The data presented in this study are available on request from the corresponding author and the first author.

## Supplementary Materials

The following supporting information can be downloaded at https://www.mdpi.com/article/10.3390/polym15132764/s1, Figure S1: DSC thermograms of cooling scan of neat PGA and modified PGA (a) neat PGA, (b) PGA_0.3, (c) PGA_0.6, (d) PGA_0.9; Figure S2: DSC thermograms of subsequent heating scan of neat PGA and modified PGA (a) neat PGA, (b) PGA_0.3, (c) PGA_0.6, (d) PGA_0.9; Figure S3: Typical tensile strain-stress curves of neat PGA and modified PGA; Figure S4: Typical flexural load-deformation curves of neat PGA and modified PGA.
