# Supplementary material for: Influence of a Multiple Epoxy Chain Extender on the Rheological Behavior, Crystallization, and Mechanical Properties of Polyglycolic Acid
Source: Polymers (Basel). 2023 Jun 21;15(13):2764. doi: 10.3390/polym15132764 (PMC10346693; doi:10.3390/polym15132764)
Supplement: Supplementary file 1 [file polymers-15-02764-s001.zip › polymers-2405869-supplementary.pdf]

Supplementary Materials

Influence of a Multiple Epoxy Chain Extender on the Rheological Behavior, Crystallization, and Mechanical Properties of Polyglycolic Acid

Jianfeng Gao [1,†], Kai Wang [1,†], Nai Xu [1,*], Luyao Li [1], Zhao Ma [1], Yipeng Zhang [1], Kun Xiang [1], Sujuan Pang [2], Lisha Pan [3], Tan Li [4]

[1] School of Materials Science and Engineering, Hainan University, Haikou 570228, China; 17889987289@163.com (J.G.); wang_kai126@163.com (K.W.); luyaoli1120@yeah.net (L.L.); taohua20042@163.com (Z.M.); zhangyipeng2013@163.com (Y.Z.); gzsfxyxk@163.com (K.X.)
[2] School of Science, Hainan University, Haikou 570228, China; psjuan@hainanu.edu.cn (S.P)
[3] School of Chemical Engineering and Technology, Hainan University, Haikou 570228, China; happylisap@hainanu.edu.cn (L.P.)
[4] Shiner National and Local Joint Engineering and Research Center, Shiner Industrial Co., Ltd., Haikou 570228, China; 13807679873@163.com (T.L.)
† These authors contributed equally to this work.
* Correspondence: xunai@hainanu.edu.cn (N.X.); Tel.: +86-1313-602-3445 (N.X.)

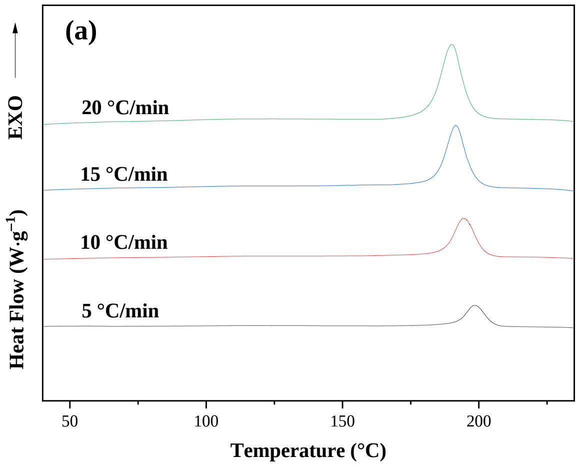

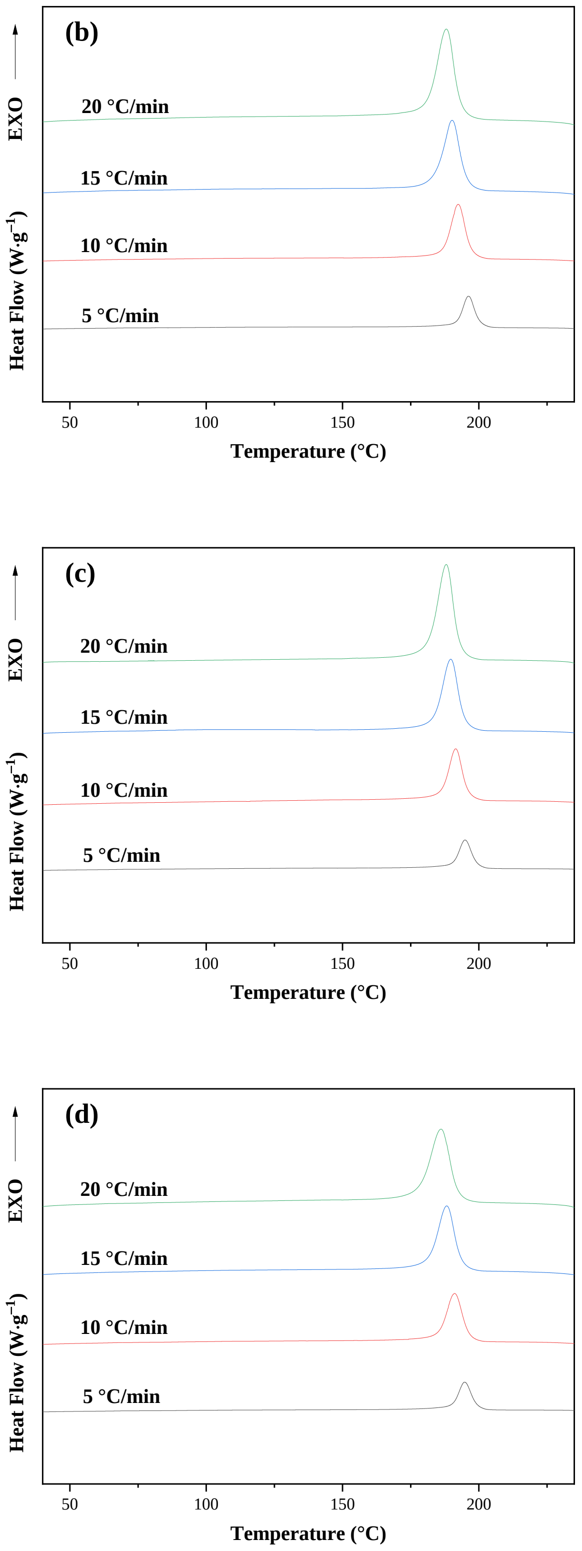

Figure S1. DSC thermograms of cooling scan of neat PGA and modified PGA (**a**) neat PGA, (**b**) PGA_0.3, (**c**) PGA_0.6, (**d**) PGA_0.9.

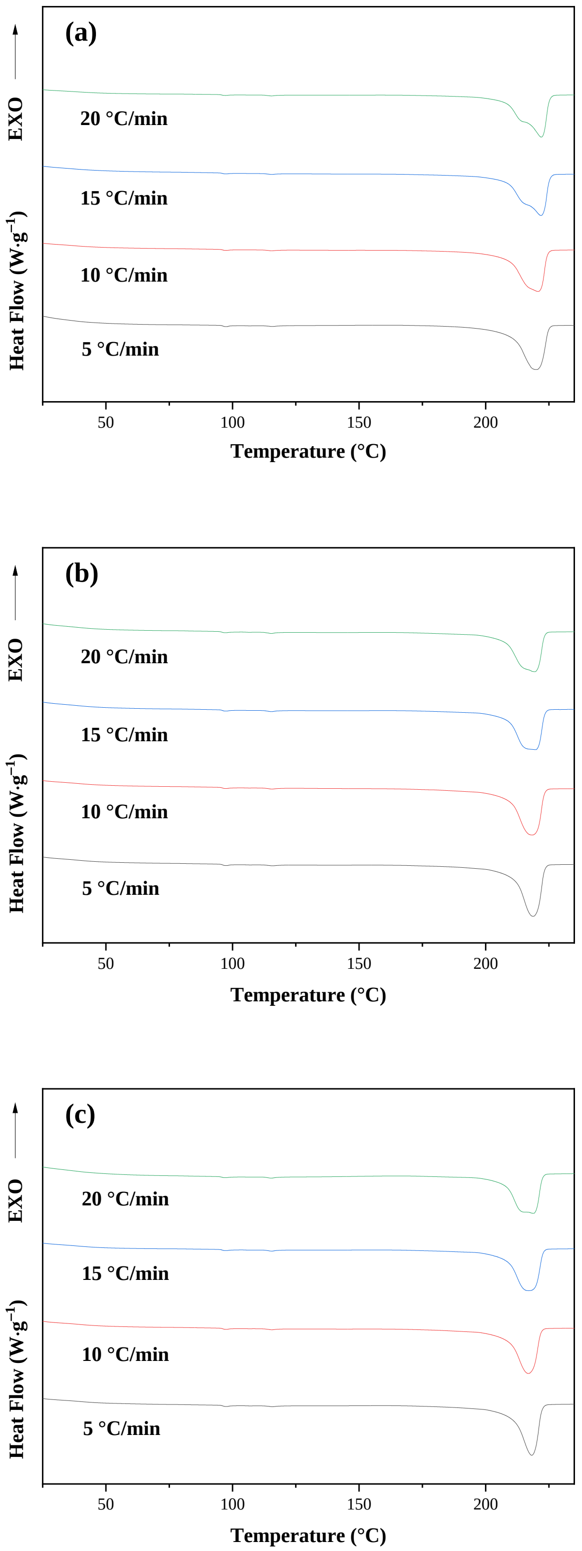

(a)
20 °C/min
15 °C/min
10 °C/min
5 °C/min
EXO
Heat Flow (W·g^{-1})
50
100
150
200
Temperature (°C)
(b)
20 °C/min
15 °C/min
10 °C/min
5 °C/min
EXO
Heat Flow (W·g^{-1})
50
100
150
200
Temperature (°C)
(c)
20 °C/min
15 °C/min
10 °C/min
5 °C/min
EXO
Heat Flow (W·g^{-1})
50
100
150
200
Temperature (°C)

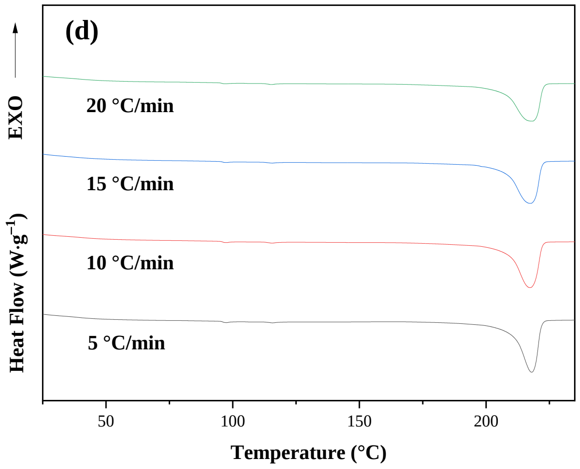

Figure S2. DSC thermograms of subsequent heating scan of neat PGA and modified PGA (**a**) neat PGA, (**b**) PGA_0.3, (**c**) PGA_0.6, (**d**) PGA_0.9.

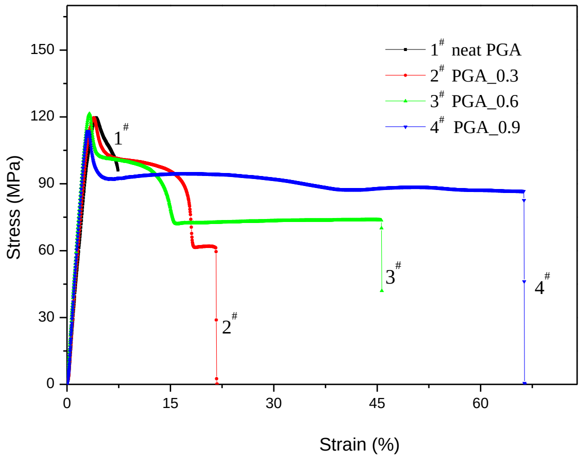

Figure S3. Typical tensile strain-stress curves of neat PGA and modified PGA.

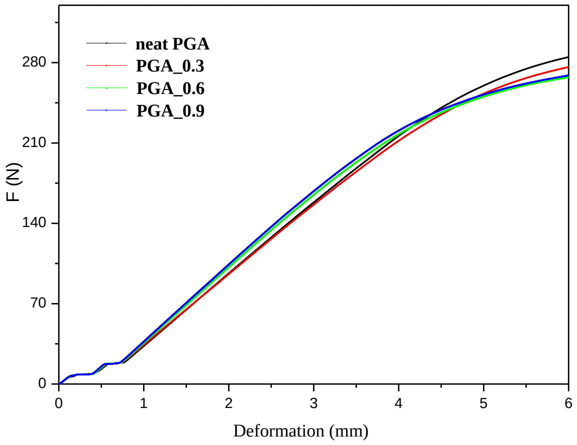

Figure S4. Typical flexural load-deformation curves of neat PGA and modified PGA.
